# Role of the glymphatic system and perivascular spaces as a potential biomarker for post‐stroke epilepsy

**DOI:** 10.1002/epi4.12877

**Published:** 2023-12-14

**Authors:** Gernot Hlauschek, John‐Paul Nicolo, Benjamin Sinclair, Meng Law, Clarissa L. Yasuda, Fernando Cendes, Morten Ingvar Lossius, Patrick Kwan, Lucy Vivash

**Affiliations:** ^1^ Division of Clinical Neuroscience, National Centre for Epilepsy, member of ERN Epicare Oslo University Hospital Norway; ^2^ The University of Oslo Oslo Norway; ^3^ Department of Neurosciences, Central Clinical School Monash University Melbourne Victoria Australia; ^4^ Department of Neurology The Alfred Melbourne Victoria Australia; ^5^ Departments of Medicine and Neurology The University of Melbourne, Royal Melbourne Hospital Parkville Victoria Australia; ^6^ Department of Radiology The Alfred Melbourne Victoria Australia; ^7^ Department of Neurology University of Campinas Campinas Brazil

**Keywords:** blood–brain barrier, glymphatic system, neuroinflammation, perivascular spaces, post‐stroke epilepsy

## Abstract

Stroke is one of the most common causes of acquired epilepsy, which can also result in disability and increased mortality rates particularly in elderly patients. No preventive treatment for post‐stroke epilepsy is currently available. Development of such treatments has been greatly limited by the lack of biomarkers to reliably identify high‐risk patients. The glymphatic system, including perivascular spaces (PVS), is the brain's waste clearance system, and enlargement or asymmetry of PVS (ePVS) is hypothesized to play a significant role in the pathogenesis of several neurological conditions. In this article, we discuss potential mechanisms for the role of perivascular spaces in the development of post‐stroke epilepsy. Using advanced MR‐imaging techniques, it has been shown that there is asymmetry and impairment of glymphatic function in the setting of ischemic stroke. Furthermore, studies have described a dysfunction of PVS in patients with different focal and generalized epilepsy syndromes. It is thought that inflammatory processes involving PVS and the blood–brain barrier, impairment of waste clearance, and sustained hypertension affecting the glymphatic system during a seizure may play a crucial role in epileptogenesis post‐stroke. We hypothesize that impairment of the glymphatic system and asymmetry and dynamics of ePVS in the course of a stroke contribute to the development of PSE. Automated ePVS detection in stroke patients might thus assist in the identification of high‐risk patients for post‐stroke epilepsy trials.

**Plain Language Summary:**

Stroke often leads to epilepsy and is one of the main causes of epilepsy in elderly patients, with no preventative treatment available. The brain’s waste removal system, called the glymphatic system which consists of perivascular spaces, may be involved. Enlargement or asymmetry of perivascular spaces could play a role in this and can be visualised with advanced brain imaging after a stroke. Detecting enlarged perivascular spaces in stroke patients could help identify those at risk for post‐stroke epilepsy.

## Graphical abstract

Stroke often leads to epilepsy and is one of the main causes of epilepsy in elderly patients, with no preventative treatment available. The brain's waste removal system, called the glymphatic system which consists of perivascular spaces, may be involved. Enlargement or asymmetry of perivascular spaces could play a role in this and can be visualized with advanced brain imaging after a stroke. Detecting enlarged perivascular spaces in stroke patients could help identify those at risk for post‐stroke epilepsy.

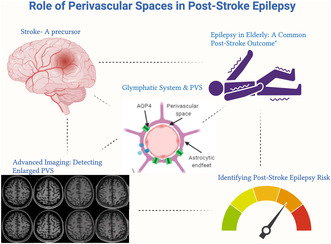

**Key points**

- Post‐stroke epilepsy is the most common cause of acquired epilepsy in the elderly and negatively affects disability and mortality rates.
- The glymphatic system is important for the drainage of interstitial fluid and waste clearance of the brain and hypothesized to play a role in several neurological conditions.
- Asymmetry of perivascular spaces is thought to reflect an impairment of glymphatic function in patients with stroke and focal epilepsy.
- Perivascular spaces can be detected on MRI using automated methods and thus could be a biomarker for post‐stroke epilepsy.

## 1 INTRODUCTION

### 1.1 Post‐stroke epilepsy

Stroke is one of the most common causes of mortality and morbidity. Acute ischemic strokes (AIS) account for >85% of strokes, the remaining being hemorrhagic.^1^ Alongside the primary injury, stroke confers an increased risk of several comorbid conditions, including the development of post‐stroke epilepsy (PSE). PSE negatively affects the long‐term functional outcome after a stroke, resulting in a higher mortality and disability rate seen in stroke patients with seizures versus stroke patients without seizures.^2^ PSE accounts for 11% of all epilepsy, 22% of cases of status epilepticus, and 55% of seizures in the elderly.^3^

### 1.2 Definitions

According to the International League Against Epilepsy (ILAE), PSE is defined as: One unprovoked (late) seizure after a stroke and a probability of further seizures, which is similar to the general recurrence risk (at least 60%) after two unprovoked seizures in patients without a brain lesion, occurring over the next 10 years.^4^ Acute symptomatic seizures are defined as occurring within 1 week after stroke onset, whereas unprovoked seizures occur more than 1 week after stroke.^5^

### 1.3 Risk factors

The cumulative risk of experiencing a seizure post‐stroke varies considerably, ranging from 2%–33% for acute symptomatic seizures and 3%–67% for unprovoked seizures in patients with ischemic stroke on which the following review is focused on.^6^, ^7^ For hemorrhagic stroke, the risk for unprovoked seizures varies from 5% to 27% according to Passero et al.^8^ Furthermore, the Cave score as a predictive instrument for epilepsy after intracerebral hemorrhage has been developed, which consists of a score between 0 and 4 points and includes the variables Cortical location, (younger) Age, stroke Volume, and Early seizures. Four points are consistent with a 46% risk of late seizures.^9^

The discrepancies in risk for PSE may be due to heterogeneity in the patient populations studied; however, better characterization of the risk factors that are associated with post‐stroke seizures will lead to greater understanding and improved prediction of who will develop post‐stroke seizures and/or epilepsy.

The SeLECT score has been suggested as a method of predicting the risk of developing PSE.^10^ It is based on five clinical predictors (severity of stroke, large artery atherosclerotic etiology, early [acute symptomatic] seizures, cortical involvement, and territory of middle cerebral artery involvement), which give a score between 0 (low risk) and 9 (high risk), where 1 point refers to an increased risk of PSE by a hazard ratio of 1.8 (95% CI 1.6–2.1; *P* < 0.0001). The SeLECT score of 0 points corresponds to a projected risk of 0.7% (with a 95% confidence interval of 0.4%–1.0%) for experiencing PSE within the first year following a stroke, and a 1.3% risk (with a 95% confidence interval of 0.7%–1.8%) within 5 years. Conversely, a SeLECT score of nine points indicates a considerably higher risk, with a 63% probability (95% CI 42%–77%) of PSE within the first year and an 83% probability (95% CI 62%–93%) within 5 years following a stroke.^10^

Other risk factors increasing the risk of developing PSE after AIS are a low Alberta Stroke Program Early CT (ASPECT) score, hemorrhagic transformation, and greater stroke volume, whereas the implementation of a code‐stroke system reduces the risk of PSE.^11^, ^12^, ^13^

### 1.4 Predictive biomarkers of PSE

Contradictory evidence exists as to whether electroencephalographic (EEG) findings in the AIS setting predict PSE. One study showed that an early EEG demonstrating background asymmetry or interictal epileptiform activity independently predicted PSE within the first year post‐stroke.^14^ However, another study found early EEG abnormalities were not found to be an independent predictor of PSE after correcting for potential confounders.^15^

Blood biomarkers may be predictors for PSE. A combination of neuroinflammatory markers released during the acute phase of an AIS consisting of low levels of S100b and Hsc‐70 and increased endostatin post‐stroke independently increases the risk of PSE by 17%.^16^

S100b, part of the DAMP protein family, is released during post‐stroke neuroinflammation, leading to microglial activation and inflammation. However, early S100b reduction after stroke can destabilize the blood–brain barrier, impacting microglial activation and contributing to post‐stroke epilepsy (PSE).^16^ Hsc70, a protective factor during stress, maintains synaptic function and protein balance.^17^ Lower Hsc70 levels in PSE‐prone stroke patients may impair the blood–brain barrier indicating its potential anti‐inflammatory role.^16^ Endostatin, an inhibitor of neurogenesis, is found at higher levels in epilepsy patients. Following brain injury, elevated endostatin levels hinder neuronal repair mechanisms.^16^, ^18^

Three studies investigated genetic biomarkers to predict post‐stroke epileptogenesis (PSE). ALDH2 rs671 polymorphism, a loss‐of‐function mutation, is linked to a higher PSE risk due to increased reactive aldehyde levels and oxidative stress, promoting inflammation.^19^ Meanwhile, CD40 ‐1C/T polymorphism, a gain‐of‐function mutation, is associated with elevated plasma sCD40L levels, implicated in inflammation, atherosclerosis, and thrombosis, increasing the risk of cerebral infarction.^20^ Additionally, the TRPM6 polymorphism (rs2274924), a loss‐of‐function mutation affecting a Mg2+ transport regulator, results in reduced extracellular Mg2+ levels, raised intracellular Na+, and intracellular Ca2+ overload, making it another genetic predictor for PSE.^21^

There is relatively little information on imaging biomarkers as predictors for PSE. In addition to those in the SeLECT score, other imaging markers that are associated with PSE include studies involving MRI and CT scans that suggest that stroke volume >70 mL is a predictor for PSE.^1^, ^22^, ^23^, ^24^

### 1.5 The glymphatic system/perivascular spaces

**FIGURE 1:**
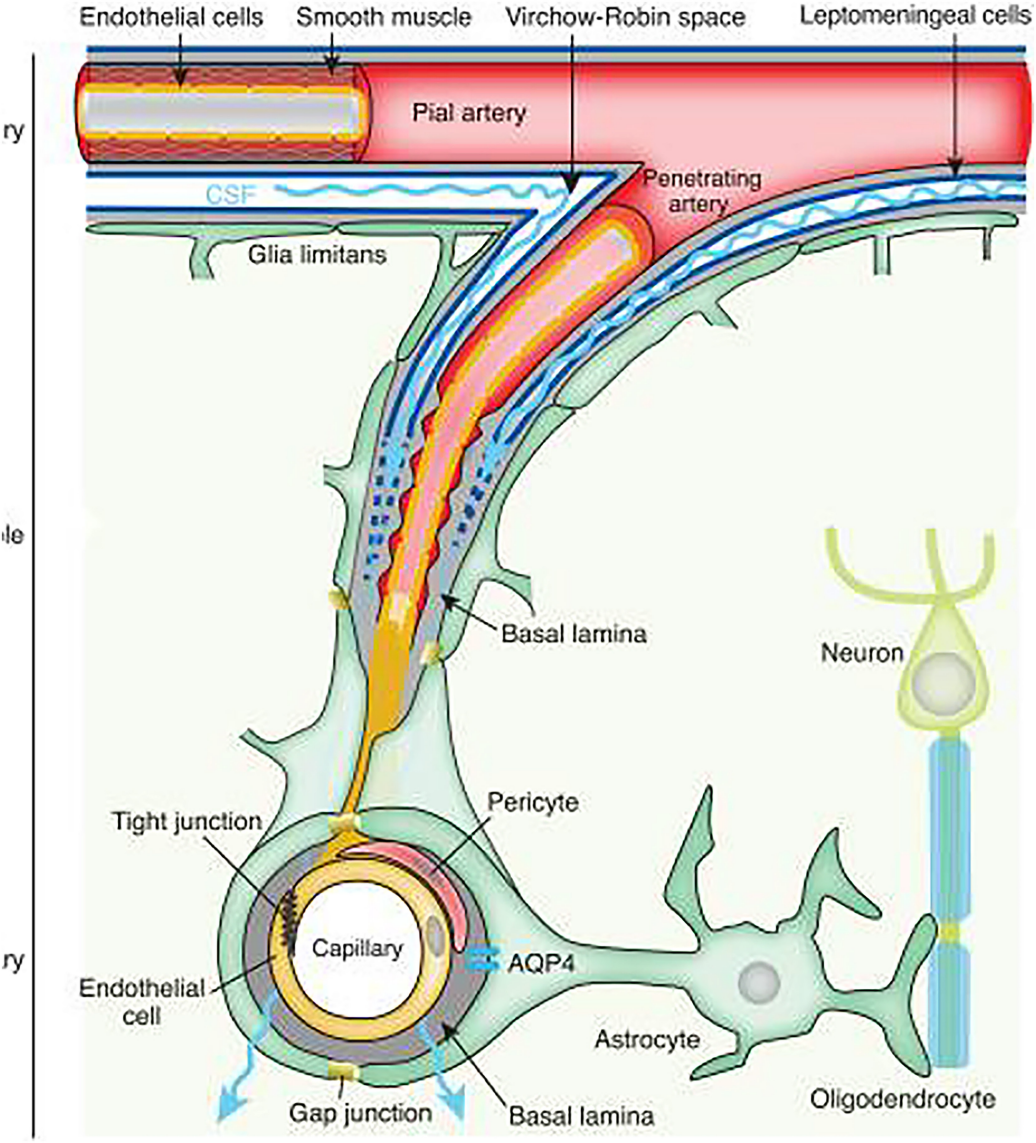
This figure illustrates the neurovascular unit that permits bidirectional exchange between microvessels and neurons. The unit consists of arteries in the subarachnoid space, which penetrate into the brain parenchyma, surrounded by CSF, forming the perivascular space (PVS) as much as neurons and astrocytes. PVSs extend from arterioles to capillaries to venules and are shaped by the basal lamina's extracellular matrix, which allow a continuity of the fluid space between arterioles and venules. The boundary of the PVSs is formed by astrocytic endfeet, expressing aquaporin‐4 (AQP4).^26^

The brain does not have lymphatic vessels, and waste clearance is primarily facilitated by the glymphatic (glial‐lymphatic) system, interstitial (ISF), and cerebrospinal fluid (CSF).^25^ The glymphatic system includes perivascular spaces (PVS), lining the blood vessels in the brain, which aid the flow of ISF and the exchange of ISF and CSF, which itself has a role in waste clearance (Figure 1).^26^ Although still under debate as to whether other mechanisms of waste clearance exist through which ISF returns to circulation through exchange across the blood–brain barrier (BBB), substantial evidence exists that PVSs are essential for the drainage of ISF.^27^ PVSs are small areas filled with CSF between blood vessels and the pia mater.^26^ From the subarachnoid space, CSF is driven into the arterial PVSs by a combination of arterial pulsality, respiration, and CSF pressure gradients. The loose fibrous matrix of the PVS can be viewed as a low‐resistance highway for CSF influx.^26^

CSF movement into the parenchyma drives convective ISF fluxes within the tissue toward the perivenous spaces surrounding the large deep veins.^26^ The ISF is collected in the perivenous space from where it drains out of the brain toward the cervical lymphatic system^26^ (Figure 1).

In many neurological and neurodegenerative conditions, it is increased PVS volume and numbers, which are positively correlated with pathology and disease.^28^, ^29^, ^30^, ^31^, ^32^ In Alzheimer's disease, Parkinson's disease, vascular dementia, cerebral amyloid angiopathy, and multiple sclerosis (MS), enlarged PVSs (ePVSs) are associated with length of disease and disease burden.^28^, ^29^, ^30^, ^31^, ^32^ Evidence to support these findings comes from the established knowledge of increased white matter hyperintensities (WMH), decreased cerebral blood flow, and increased BBB permeability all of which may contribute to the obstruction of CSF clearance and dilation of CSF spaces/enlargement of PVS.^33^

However, it is currently unknown how WMH affect PVS enlargement. Not only that but most studies have been limited to small sample sizes and have not been widely replicated between and across diseases. Furthermore, these studies are typically cross‐sectional in design, providing only a snapshot in time and lack measuring PVS dynamics over time in relation to the course of disease.^32^, ^34^, ^35^

### 1.6 The role of imaging

Imaging of ePVS has become increasingly common over recent years. Early approaches in humans have studied glymphatic clearance using contrast‐enhanced MRI in patients with idiopathic normal pressure hydrocephalus compared to healthy individuals, visualizing intrathecally applied gadobutrol clearance.^36^ Less invasive approaches with intravenous gadolinium have also been applied.^37^ However, both methods are time‐consuming, carry the risk of allergy, and are contraindicated in patients with renal impairment.

Diffusion tensor imaging along the perivascular space (DTI‐ALPS) has been applied as a non‐invasive method to assess the diffusion capacity of water along ePVS at regions of the brain where white matter fiber tracts and PVS are perpendicular.^38^ Unfortunately, this method is currently limited to one region of interest in the brain where the medullary arteries and veins intersect with the ventricular wall, and it further requires co‐registration of susceptibility‐weighted imaging (SWI) with DTI, rendering it more inaccurate in elderly patient populations.^38^

With advanced MRI technology, both visual rating and automated methods of detecting ePVS become more accessible. Wardlaw et al. developed a visual rating scale involving manually counting ePVS on selected slices of T2‐weighted images (with T1 and FLAIR being available) in three different areas of the brain (centrum semiovale, basal ganglia, and midbrain), which are then assigned to four different categories according to the number of PVS in each slice.^39^ However, this method is time‐consuming, carries a considerable risk of detection bias, and has limited application for longitudinal studies due to the categorical nature of the visual rating scale, which renders the chance of detecting changes in ePVS burden within the same category impossible.

**FIGURE 2:**
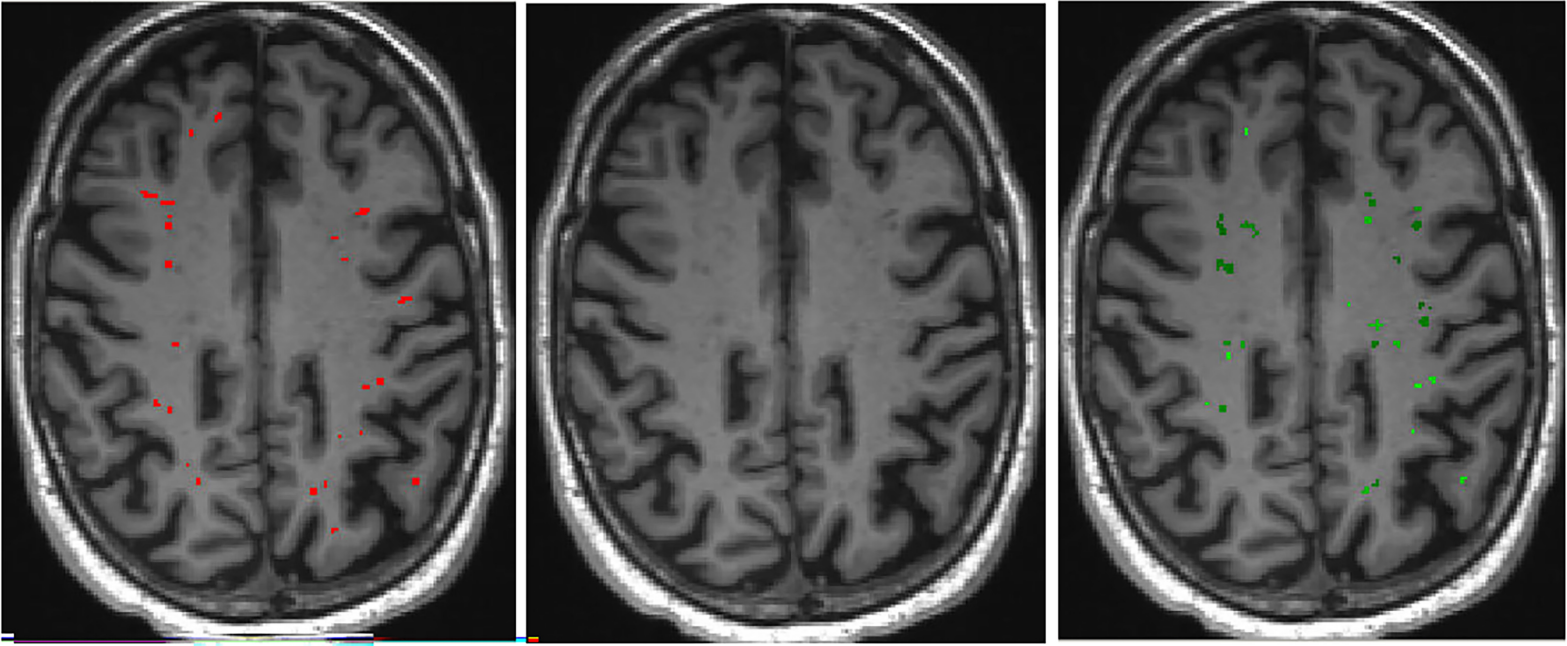
MRI T1‐weighted images that show ePVS burden in a patient with behavioral variant fronto‐temporal dementia. The left image shows manual segmentation of ePVS highlighted in red. In the right image, automated segmentation of ePVS using the Multimodal Autoidentification of Perivascular Spaces (MAPS) algorithm is highlighted in green with a slightly higher number of ePVS (27 vs. 24) becoming visible using MAPS. However, the manual counts and automated segmentations show a weak correlation, likely due to poor image quality and motion artifacts. The dataset's scans resemble typical clinical practice quality, featuring low contrast‐to‐noise ratios (CNR). MAPS performs better with higher‐quality datasets boasting higher CNR. Thus, future research should optimize ePVS segmentation for clinical practice‐standard scan qualities.^46^

Automated ePVS detection methods are currently emerging which carry the advantages of being able to objectively quantify standardized whole‐brain assessment of ePVS with low risk of bias and higher sensitivity and specificity^40^ (Figure 2). In particular, these methods can be divided into three main categories consisting of intensity thresholding, vesselness filtering, and machine learning.^41^, ^42^, ^43^ In addition to providing more accurate information on ePVS count, they also enable the measure of ePVS length, width, and volume.^44^ For further details, we refer to a newly published comprehensive review by Moses et al.^45^ Advances in both manual and automated ePVS detection methods have widened the investigation of PVS as a biomarker of disease across a range of neurological diseases, enabling quantification from standard clinical MRI protocols with lower quality images than research scans due to motion artifacts (Figure 2).^46^ However, there is still need to optimize ePVS segmentation for typical clinical practice scan qualities.^40^ Another consideration is that current imaging methods cannot discriminate between venous and arterial ePVS. However, right now we do not know whether there is any difference from a pathological point of view.

### 1.7 ePVS and stroke

Animal studies with intrathecally administered gadolinium and contrast‐enhanced MRI demonstrated that the glymphatic system is severely impaired after AIS: In one study, reduced gadolinium clearance at 3 h but not at 24 h after an AIS reflects disturbed glymphatic function in the acute phase post‐stroke.^47^ Another study showed ipsilateral delayed gadolinium clearance in two areas of reference (substantia nigra and ventral thalamic nucleus) at Day 1 and Day 7 post‐stroke, representing impaired glymphatic function in remote secondary degenerative areas after AIS.^48^

However, a meta‐analysis including five studies with lacunar versus non‐lacunar ischemic stroke and two studies with acute ischemic stroke did not find any association between ePVS and ischemic stroke.^49^

In the setting of an AIS, CSF circulation is impaired.^50^ However, there is lack of consensus on the dynamics. On the one hand, it is postulated that rapid depolarization caused by brain ischemia leads to ePVS and increased glymphatic flow, which might be one of the underlying pathological mechanisms for tissue swelling and cerebral edema.^51^ On the other hand, studies have shown decreased CSF influx in the ipsilateral cortex from stroke onset until 7 days after an AIS.^48^ Reduced glymphatic clearance then leads to the accumulation of toxic solutes and proteins such as amyloid‐beta and hyperphosphorylated tau and pro‐inflammatory cytokines within the infarcted area.^50^ Thus, impairment of the glymphatic system post‐stroke could also contribute to the increased risk of both post‐stroke dementia and post‐stroke depression.^50^, ^52^

Aquaporin 4 (AQP‐4) channels are thought to play a vital role in the impairment of the glymphatic system through involvement in several biological processes such as regulation of brain edema, promotion of migration of astrocytes, calcium signal transduction, and synaptic plasticity. AQP‐4 channels are upregulated after stroke and may contribute to increased cerebral edema post‐stroke.^53^, ^54^ On the other hand, the downregulation of AQP‐4 in animal models resulted in aggravated brain damage through the hypertrophy of astrocytes and loss of hippocampal neurons.^55^ Finally, redistribution of AQP‐4 channels from peri‐vessel locations to the parenchyma might enhance brain edema and impair glymphatic function.

Stroke patients suffer from an abnormal pattern of melatonin release in conjunction with premature and dysfunctional release of cortisol, which is thought to be responsible for the disruption of their circadian rhythm.^56^ Sleep is important for glymphatic clearance, and PVS are reduced during wakefulness leading to decreased clearance of metabolic waste products such as amyloid‐beta and other neurotoxic proteins.^57^ During sleep, PVS enlarge, providing greater clearance of toxic proteins and waste products. A major mediator of this mechanism is thought to be norepinephrine, which is increased during wakefulness and associated with the suppression of glymphatic function.^57^ Hence, it is hypothesized that sleep disturbance in stroke patients leads to impaired glymphatic function causing accumulation of neurotoxic byproducts such as amyloid‐beta, tau, and glutamate in the interstitial and PVS.^57^

### 1.8 ePVS and epilepsy

We conducted a literature search up to February 2023 using PubMed. The electronic search was supplemented by hand searching the bibliographies of review articles and the authors' personal files. We used combinations of the following terms: glymphatics, perivascular spaces, epilepsy, seizures, stroke, and post‐stroke epilepsy. Ten studies were found investigating the relationship between PVS as measured by MRI and epilepsy (Tables 1 and 2). Overall, ePVSs are observed in epilepsy with consistent findings of increased burden in adult epilepsies, whereas pediatric epilepsies show variability in ePVS burden, with some studies showing no difference compared to controls and a small number showing increased ePVS.^58^, ^59^, ^60^, ^61^, ^62^, ^63^, ^64^, ^65^, ^66^, ^67^ Two out of these 10 studies found an association between ePVS count and volume and seizure duration^59^, ^60^; however, to our knowledge, further evidence on the role of the glymphatic system in epileptogenesis in general is still sparse.

**TABLE 1:** ePVS as a biomarker for epilepsy in children.

References	Age group	Cases (males %) controls (males %)	Epilepsy type	Method	Finding
Boxermann et al^58^	3–12 years	Children with RE: 25 (76%), Children with Migraine: 25 (56%)	Focal: Rolandic epilepsy (RE)	Visual rating	Non‐significant *P* = N/A
Liu et al^59^	3–13 years	Children with IGE: 32 (62.5%), Healthy children: 30 (76.6%)	Idiopathic generalized epilepsy (IGE)	Automated	‐ePVS in epilepsy patients and seizure duration positively correlated with ePVS counts (*P* < 0.001) and volume (*P* = 0.001)
Salimeen et al^60^	6–60 months	Epilepsy: 33 (63.3%), SFS: 26 (61.54%), Controls: 28 (60.61%)	Simple febrile seizures (SFS) and unspecified epilepsy	Automated	‐Higher ePVS count + volume in SFS and epilepsy patients versus controls (*P* < 0.001) ‐Higher ePVS COUNT in epilepsy patients versus SFS patients (*P* < 0.001) ‐Seizure duration positively correlated with ePVS counts and volume (*P* < 0.001)
Spalice et al^61^	1–18 years	Epilepsy: 26 SFS: 4	Prolonged febrile seizures and generalized and focal epilepsy	Visual rating	Non‐significant (*P* = N/A)

Abbreviations: IGE, idiopathic generalized epilepsy; SFS, simple febrile seizures.

**TABLE 2:** ePVS as a biomarker for epilepsy in adults.

References	Cases (males %), controls (males %)	Epilepsy type	Method	Finding
Feldman et al^62^	21 (61.9%), 17 (64.7%)	Focal epilepsy	Visual rating	Higher ePVS asymmetry in epilepsy patients, which corresponds to seizure focus (*P* = 0.016)
Duncan et al^67^	15 (80%), 6 (50%)	PTE	Visual rating	TBI patients have a higher ePVS asymmetry than healthy controls (*P* = 0.001). PTE patients have highest ePVS asymmetry with less PVS detected in the affected hemisphere (*P* = N/A)
Lee et al^63^, ^64^	25 (52.0%), 26 (42.3%)	TLE with hippocampal sclerosis	Automated DTI‐ALPS	TLE patients have reduced glymphatic function/DTI‐ALPS index (*P* = 0.015)
Lee et al^63^, ^64^	109 (53.2%), 88 (53.4%)	Focal epilepsy	Automated DTI‐ALPS	No association between DTI‐ALPS index and epilepsy (*P* = 0.861) Negative correlations were found between DTI‐ALPS index and age (*P* = 0.01) in both epilepsy patients and healthy controls
Lee et al^65^	39 (61.5%), 38 (57.9%)	JME	Automated DTI‐ALPS	Reduced DTI‐ALPS index in JME patients (*P* = 0.041)
Yu et al^66^	PSE: 58 (69.0%) Non‐epilepsy AIS: 254 (59.8%), controls: 20	PSE	Visual rating	Asymmetric distribution of ePVS in the central semiovale region in PSE patients (*P* = 0.004)

Abbreviations: AIS, acute ischemic stroke; DTI‐ALPS, diffusion tensor imaging along the perivascular space; JME, juvenile myoclonic epilepsy; PSE, post‐stroke epilepsy; PVS, enlarged perivascular space; TLE, temporal lobe epilepsy.

A study of 25 children with rolandic epilepsy (RE) and 25 controls mentions ePVSs among other imaging features. However, due to low interrater agreement for visual rating, no relationship between RE and ePVS could be found.^58^

Liu et al studied 32 children with newly diagnosed idiopathic generalized epilepsy (IGE) compared to controls. An automated algorithm was used to segment the ePVS in the white matter above the ventricles. They found that children with IGE had higher ePVS counts and volume and that the seizure duration was positively correlated with ePVS counts and volume.^59^ Another study by the same group studied 26 children with simple febrile seizures (SFS), 33 children with epilepsy and 28 controls. In line with previous studies, ePVS counts were higher in the SFS and epilepsy groups and seizure duration was positively correlated to ePVS counts and volume. However, the ePVS size was lower in the SFS group than in the epilepsy group.^60^

Spalice et al. analyzed a group of 26 children with unspecified epilepsy and 4 children with SFS. ePVSs were counted visually in three different brain regions (supratentorial white matter, basal ganglia, and midbrain) and compared to a follow‐up MRI, taken at least 12 months from the initial MRI study. No significant correlation between a seizure onset zone (SOZ) and ePVSs or dynamic changes of PVSs on the follow‐up MRI was observed.^61^

Feldman et al. studied 21 patients with focal epilepsy and 17 healthy controls. Using high‐field MRI (7‐Tesla), ePVSs were visually counted to calculate an asymmetry index (AI). A threshold of AI >0.2 was defined as high asymmetry in PVS distribution. Greater ePVS asymmetry was observed in epilepsy patients and the area of maximum ePVS asymmetry corresponded to the suspected SOZ with fewer ePVSs detected ipsilateral to the SOZ.^62^

Duncan et al studied 15 patients with traumatic brain injury (TBI) and six healthy controls. TBI patients have a higher asymmetry than healthy controls; furthermore, five TBI patients developed PTE, which were among the six TBI patients with highest asymmetry. The affected hemisphere after TBI and potential SOZ showed a lower ePVS count compared to the unaffected one.^67^

Both studies are suggestive of epilepsy causing glymphatic dysfunction on the epileptogenic side with ePVS asymmetry associated with reduced ePVS count on the affected side.^62^, ^67^

Park et al. utilized DTI‐ALPS for visualizing glymphatic system dysfunction in three different epilepsy patient cohorts: Firstly, a study of patients with temporal lobe epilepsy (TLE) and hippocampal sclerosis found reduced glymphatic function in epilepsy patients compared to controls.^63^ Furthermore, the group analyzed 109 patients with newly diagnosed focal epilepsy and 88 controls. The DTI‐ALPS index was compared to other DTI connectivity parameters, and a significant correlation was found in both groups, indicating that low structural connectivity correlates with glymphatic system dysfunction and age. However, there was no association between DTI‐ALPS index and epilepsy.^64^ Finally, they studied patients with juvenile myoclonic epilepsy (JME, n = 39) and controls finding a reduced DTI‐ALPS index in JME, which also correlated negatively with age.^65^

### 1.9 Enlarged perivascular spaces, vascular risk factors, and post‐stroke epilepsy

Studies have revealed a notable link between ePVS and cerebral small vessel disease (CVD). Particularly, previous lacunar strokes and CVD are found to be independent predictors of ePVS burden, regardless of the patient's age.^68^ On the other hand, there is an association between white matter hyperintensities (WMH), CVD, and large artery atherosclerosis,^69^ implying that there is an indirect link between ePVS and atherosclerosis. These findings suggest a complex interplay between ePVS and various vascular pathologies. Furthermore, another study confirmed these findings, highlighting associations between a higher ePVS burden, an increased prevalence of WMH, lower gray matter volume, and a heightened prevalence of cortical brain infarctions.^70^ These connections emphasize the intricate relationship between ePVS and the broader spectrum of cerebral vascular health.

Furthermore, research has uncovered a specific connection between ePVS and the risk of stroke recurrence. A high burden of ePVS in the basal ganglia of the brain appears to be associated with an increased likelihood of recurrent strokes.^71^ Notably, both the centrum semiovale and the basal ganglia receive substantial vascularization from the middle cerebral artery (MCA).

Exploring the link between ePVS and PSE, a study by our group focused on 312 patients with acute ischemic stroke (AIS), of which 58 later developed PSE. This study observed an asymmetric distribution of ePVS in the centrum semiovale region in patients with PSE, but not in the AIS control group. However, it is important to note that the methods employed in this study did not include laterality analysis, leaving the true asymmetry between the ipsi‐ and contralateral hemispheres yet to be conclusively demonstrated.^66^ No link has been found between acute symptomatic seizures and ePVS.

It is essential to assess ePVS both post‐stroke and ideally before the first seizure occurs. In this context, it is suggested to measure ePVS in the entire brain and in specific regions of interest categorized by both anatomical divisions (such as the centrum semiovale, basal ganglia, and midbrain) and vascular territories (including the anterior cerebral artery, middle cerebral artery, and posterior cerebral/vertebral artery).

Overall, evidence today is yet still in the sphere of detecting and defining ePVS in disease. Understanding of ePVS across the lifespan is still limited. Most cross‐sectional studies report an increase of ePVS numbers and volume with increasing age.^72^, ^73^ However, one particular study including healthy adults from the human connectome project^74^ across the lifespan concludes with linearly increasing ePVS volume in white matter with age, but declining numbers from middle age.^75^ This is a surprising finding and indicates the relevance to further investigate ePVS dynamics in disease.

The following are pathophysiological theories related to ePSE and PVS, which need further investigation in the next 3–5 years.

## 2 PATHOPHYSIOLOGICAL THEORIES

### 2.1 Epileptogenesis and stroke

Epileptogenesis refers to the process in which brain networks are modified to generate spontaneous recurrent seizures. Epileptogenesis can also be considered the pathogenic mechanisms that produce disease progression in terms of frequency and severity of seizures once an epilepsy diagnosis has been made.^76^ On a cellular and network basis, it is postulated that network instability toward excitatory positive feedback loops and a decrease in inhibitory feedback, sprouting of new synaptic connections between neurons after brain injury, and changes in the transcriptome play a major role during epileptogenesis.^76^, ^77^, ^78^ The latter occurs mainly through epigenetic modifications such as DNA methylation, histone modification, and transcriptional regulation by micro‐RNAs.^79^, ^80^

Epileptogenesis post‐AIS occurs mainly in patchy microlesions in cortical regions of the penumbra or peri‐infarct area.^81^ In these zones, it is thought that through increased BBB permeability, excitotoxicity, and post‐stroke transcriptional changes, an immune response is triggered, leading to neuronal plasticity and the development of epileptogenic networks. Stroke carries a disruption of the BBB, being a driving force for neuroinflammation and excitotoxicity.^81^, ^82^

### 2.2 The role of ePVS in epileptogenesis

In patients with focal epilepsy, ePVS asymmetry is correlated with the suspected SOZ.^62^ However, the role of altered ePVSs in the development of PSE remains unknown. Recently, ePVS have been shown to be associated with cognitive impairment after stroke and transient ischemic attack.^83^ We hypothesize that in the setting of an AIS, the occlusion of PVS contributes to the accumulation of neurotoxic metabolic byproducts in the penumbra that lead to seizures and the development of epilepsy.

We postulate that the glymphatic system is involved in epileptogenesis through three main mechanisms:

1. Inflammation: The release of pro‐inflammatory mediators, dysfunction of endothelial tight junction, abnormal CSF‐ISF exchange and poor glymphatic function, impaired PVS, and the entrance of leukocytes releasing pro‐inflammatory markers results in further breakdown of BBB structures.

Seizures are thought to be associated with decoupling of the neurovascular unit and BBB dysfunction at the arteriolar and capillary levels.^84^ Furthermore, BBB permeability leads to homeostatic and inflammatory dysregulation and abnormal neuronal transmission, which in turn increases seizure activity, leading to a vicious cycle and bidirectional relationship between seizure activity and BBB disruption.^85^

Acquired brain injuries such as an AIS are known to disrupt the BBB, further exacerbating the positive feedback loop between seizures and BBB disruption. BBB disruption is one of the major driving forces for neuroinflammation through the accumulation of immune cells such as T‐ and B‐lymphocytes and cytokines such as interleukin‐1b (IL‐1b), interleukin‐6 (IL‐6), and tumor necrosis factor‐alpha (TNF‐alpha) in the PVS leading to glymphatic impairment, which then aggravates neuroinflammation by suppressing cytokine clearance from the brain.^86^, ^87^

Neuroinflammation in turn exacerbates glymphatic dysfunction through different mechanisms:

First, inflammatory cytokines and upregulated major histocompatibility complex via the Toll‐like receptor 4 pathway, by induction of the transcription factor NF‐kb, lead to CSF hypersecretion by the activation of the Na+/K+/Cl‐ co‐transporter in the choroid plexus and secretion of pro‐inflammatory cytokines IL‐1b, IL‐6, and TNF‐alpha.^88^ Enhanced clearance of antigens via the glymphatic system to the meningeal lymphatic system then upholds the immune response by attracting peripheral immune cells to the meningeal lymphatic system.^88^

Additionally, accumulation of perivascular immune cells and cytokines leads to impairment of glymphatic flow. The latter are both drivers of additional inflammation, leading through activation of microglia to astrogliosis and further dysregulation of AQP‐4 expression.^89^ AQP‐4 expression during inflammation is upregulated; however, loss of vascular AQP‐4 polarization leads to decrease of glymphatic flow, because the additional AQP‐4 channels are inserted in the cell body and peri‐synaptic processes of astrocytes, rather than in their vascular endfeet, which sheath the PVS.^72^

Finally, sustained inflammation and chronic presence of cytokines lead to further BBB breakdown, thus upholding the vicious cycle and bidirectional relationship between seizure activity, BBB disruption, neuroinflammation, and glymphatic dysfunction.

2 Seizure activity: There is a bidirectional relationship between glutamate release and seizures, leading to the overactivation of endothelial cells and increased amyloid and tau production, thus impairing glymphatic function.

It is known that seizures result in neuronal depolarization leading to increased levels of glutamate in the synapses and initiation of excitotoxicity, where NMDA and AMPA receptors are overstimulated, leading to the increase of intracellular Na+/Cl− and Ca2+. Increased intracellular Na + leads to acute swelling of the neuron, whereas increased intracellular Ca2+, through activation of different catabolic events, leads to neuronal degeneration and is further responsible for additional glutamate release through a positive feedback loop.^90^ Particularly, in the setting of AIS, it is postulated that glutamate accumulates in the penumbra leading to excitotoxicity, neuronal depolarization, and seizures in a hypothesized bidirectional relationship.

**FIGURE 3:**
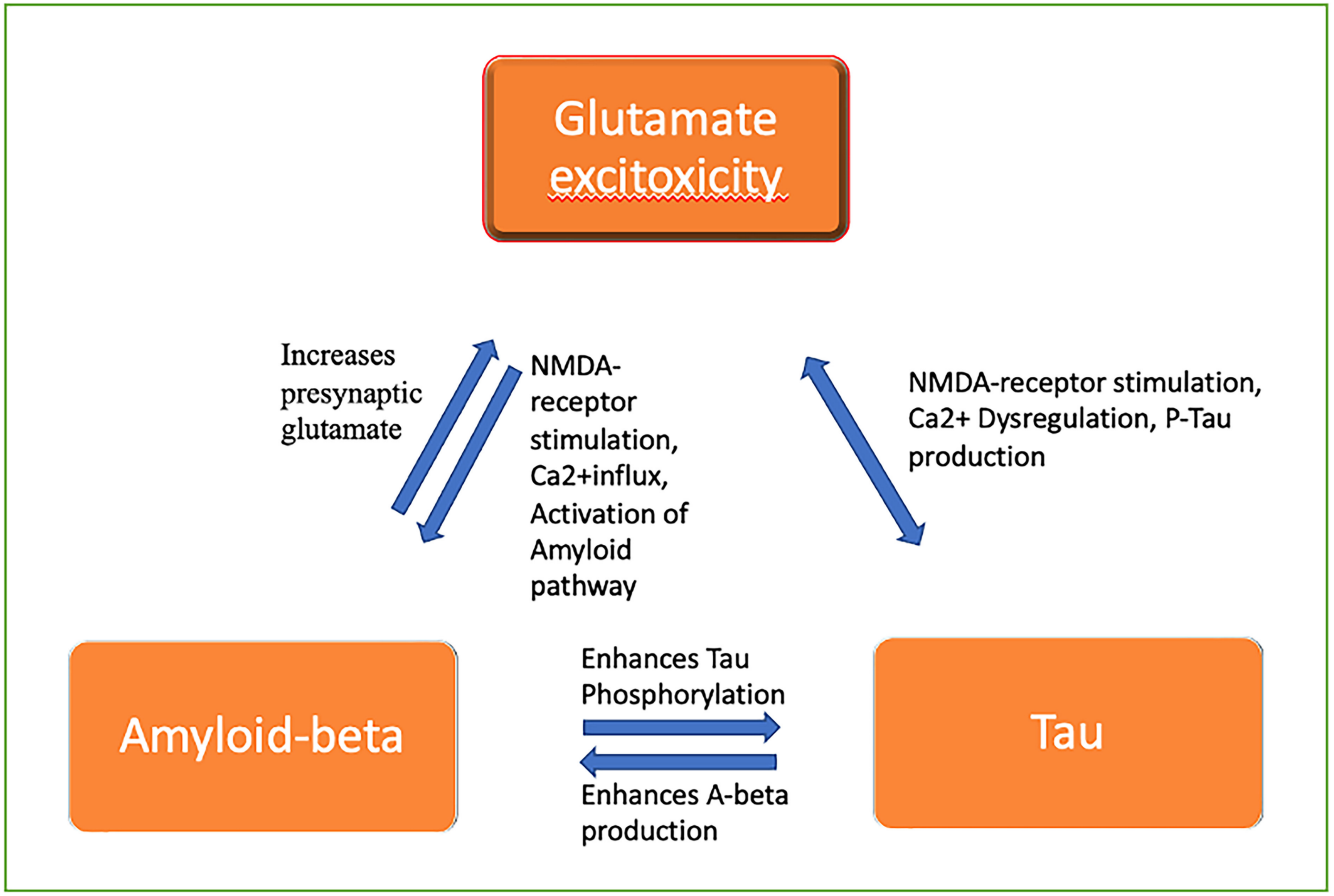
The figure visualizes the hypothesized bidirectional relationships and positive feedback mechanisms between glutamate, amyloid‐beta, and tau whereby amyloid‐beta increases presynaptic glutamate, further exacerbating excitotoxicity, and increasing accumulation of both amyloid‐beta and tau. Amyloid‐beta and tau are in a bidirectional relationship whereby increases in amyloid‐beta increase phosphorylated tau and vice versa. Meanwhile, phosphorylated tau also increases NMDA‐mediated receptor stimulation and excitotoxicity.

Moreover, there is a further hypothesized bidirectional relationship between amyloid‐beta‐mediated toxicity, glutamate‐mediated excitotoxicity, and aggregation of intracellular neurofibrillary tangles of phosphorylated tau (p‐tau): Firstly, amyloid‐beta increases presynaptic glutamate release, inhibiting clearance of glutamate leading to increased intracellular NMDA receptor‐mediated Ca2+ influx, which in turn enhances amyloid‐beta production through activation of amyloidogenic and non‐amyloidogenic pathways and increased amyloid‐precursor protein processing.^91^ Second, increased extracellular amyloid‐beta can induce intracellular tau phosphorylation, and p‐tau in reverse enhances the amyloid‐beta production.^91^ Furthermore, overstimulation of NMDA receptors increases p‐tau production through dysregulation of intracellular Ca2+‐mediated pathways^91^, ^92^, ^93^ (Figure 3).

**FIGURE 4:**
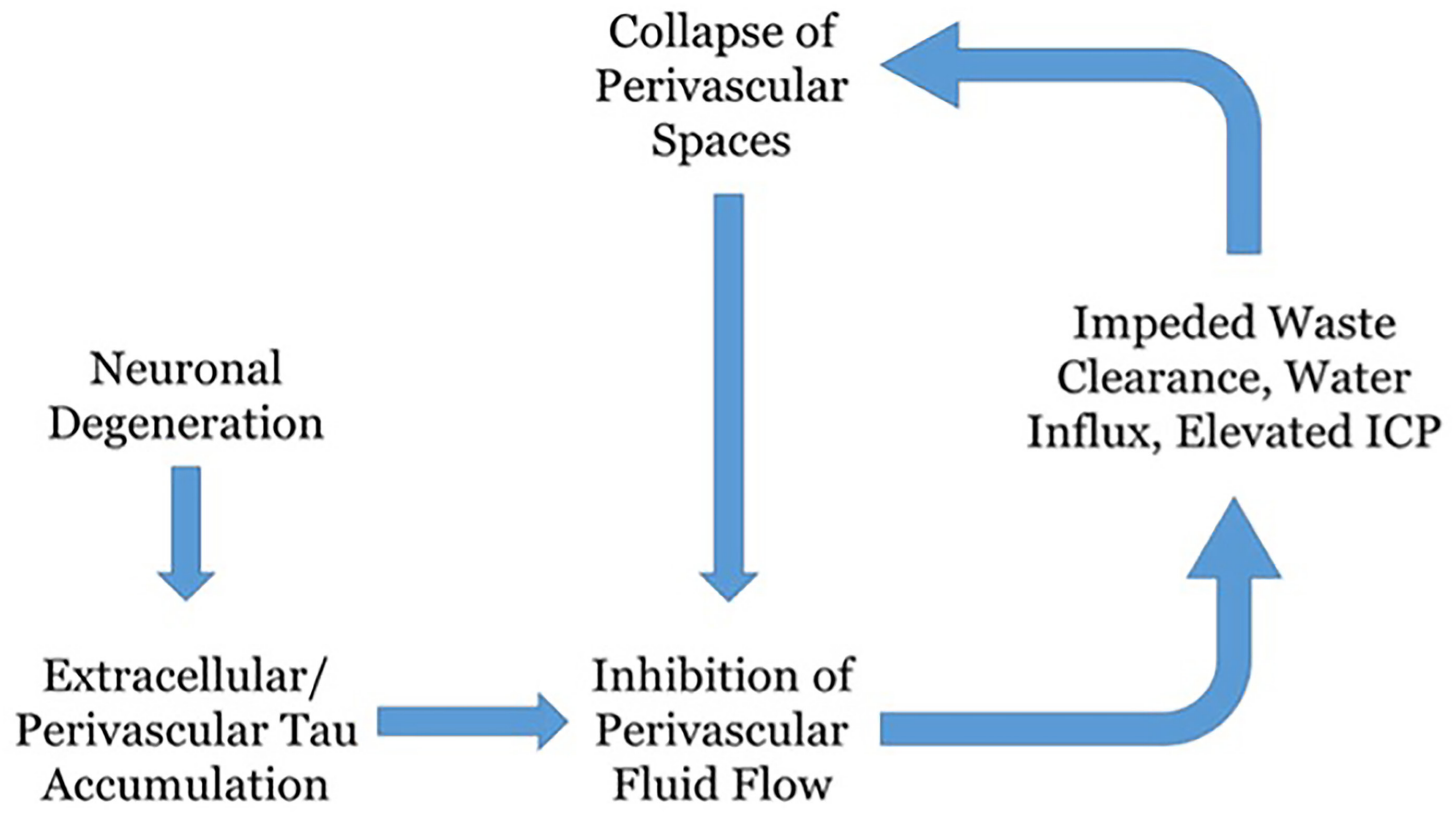
The impairment of the glymphatic system by neurotoxic waste products: This figure highlights the pathway of neurodegeneration and how excess tau leads to the collapse of PVS.^107^

Thus, aggregation of neurotoxic waste products such as p‐tau and amyloid‐beta in the PVS impedes glymphatic clearance through the blockage of PVS. Glutamate overproduction, through overactivation of endothelial cells, further impairs glymphatic dysfunction, and the depolarization of neurons through Ca2+ overload increases the risk of further epileptic seizures (Figure 4).^94^, ^95^

3 Sustained hypertension: Sustained hypertension during a seizure causes weakened arterial pulsation and thus reduces fluid flow through the glymphatic system; this can be bidirectional, in that seizure activity can also evoke hypertension to reduce glymphatic flow.

It is postulated that there is a bidirectional relationship between hypertension and epilepsy.^96^ On the one hand, activation of the renin–angiotensin system triggered by the sympathetic nervous system causes cerebral damage and lowers the seizure threshold, whereas on the other hand, seizures are linked to increased sympathetic tone, which in turn triggers hypertension.^97^ Studies have shown that hypertension might be an independent risk factor for late‐onset epilepsy and that angiotensin receptors (AT) are overexpressed in patients with seizures; thus, treatment with AT‐receptor antagonists, e.g., losartan, might have a disease‐modifying effect in reducing seizure susceptibility.^98^ The glymphatic system might play a role in these mechanisms because it is thought to be impaired by sustained hypertension, as shown in animal studies and in humans with idiopathic intracranial hypertension (IIH).^99^, ^100^ Those studies contend that arterial hypertension induces a change in the vasculature dynamics that reduces perivascular pumping, decreasing the flow of CSF in PVS.^99^ Furthermore, in patients with IIH intrathecal gadolinium does have an increased influx but delayed clearance from brain parenchyma. The responsible mechanisms for this might be hypertension‐associated reduced intracranial compliance and reduced ventricular reflux as much as pro‐inflammatory leakage of fibrinogen, which causes astrogliosis, all three resulting in impaired glymphatic clearance, shown by gadolinium enhancement.^100^

We thus hypothesize that sustained hypertension during a seizure weakens arterial pulsatility, resulting in the decoupling of the neurovascular unit and impaired glymphatic function.^95^, ^101^

## 3 MODELING POST‐STROKE EPILEPTOGENESIS: THE ROLE OF ePVS

### 3.1 Hypotheses

1. Impairment of the glymphatic system during a stroke contributes to post‐stroke epileptogenesis.

**FIGURE 5:**
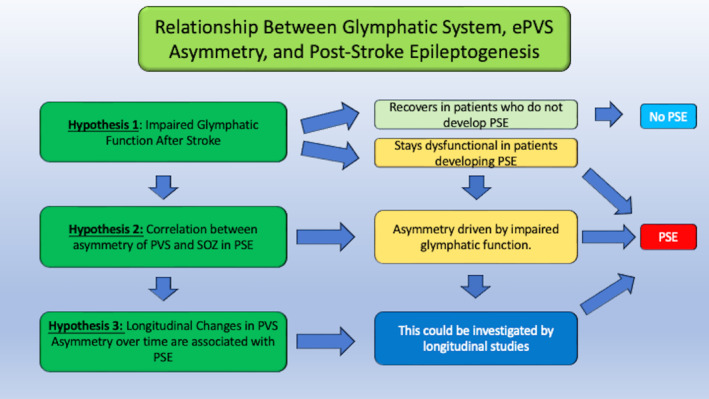
The Interplay of ePVS Asymmetry and Post‐Stroke Epileptogenesis. This figure provides a comprehensive view of the intricate relationship between enlarged perivascular spaces (ePVS) asymmetry and the development of post‐stroke epilepsy (PSE). It integrates three key hypotheses: 1. Failed Recovery of Glymphatic Clearance after a Stroke: The figure explores the hypothesis that impaired glymphatic clearance mechanisms following a stroke may contribute to ePVS formation and subsequently influence post‐stroke epileptogenesis. 2. Correlation of Asymmetry of ePVS and Seizure Onset Zone (SOZ): It highlights the correlation between the asymmetry of ePVS distribution and the localization of the SOZ, shedding light on the potential significance of ePVS patterns in the context of epilepsy development. 3. Longitudinal Changes of ePVS Over Time and Their Relationship with Post‐Stroke Epileptogenesis: The figure addresses the dynamic nature of ePVS by examining how longitudinal changes in ePVS burden may be associated with the onset and progression of post‐stroke epileptogenesis. In essence, the figure serves as a visual guide to understanding the multifaceted interplay of these hypotheses in the context of ePVS asymmetry and its relevance to PSE.

Animal studies have shown that during the acute phase after ischemic stroke glymphatic clearance is reduced between 24 h^47^ and 7 days^48^ and increases again after initial impairment. Furthermore, similar results were reproduced in a human study, which showed impaired glymphatic function measured by DTI‐ALPS following cerebral infarction ipsilateral to the stroke. However after initial impairment, DTI‐ALPS increased again after 14 days, which is suggestive of glymphatic function recovery after the acute phase of the stroke (Figure 5).^102^

As described above, three studies measuring glymphatic clearance by the functional DTI‐ALPS method demonstrated reduced glymphatic function in focal epilepsy patients compared to healthy controls. In generalized epilepsy, a positive correlation between ePVS volume and numbers and the duration of seizures has been shown, giving an indication of a relationship between the glymphatic system and epileptogenesis.^59^, ^60^ Yet, there is little further evidence on that specific relationship.

It is thus hypothesized that in those stroke patients who develop PSE, the glymphatic system does not appropriately recover after a stroke as it does in stroke patients who do not develop PSE. Hence, a glymphatic system that stays dysfunctional post‐stroke might predict unprovoked seizures (and PSE) post‐stroke.

2 There is a positive correlation between an increased asymmetry of ePVS and the development of PSE, and the asymmetry in the distribution of the ePVS has a relationship to the SOZ in patients who develop PSE.

It is hypothesized that the asymmetry is driven by impaired glymphatic clearance ipsilateral to the stroke and that the side of impaired ePVS points to the SOZ. In other words, we contend that increased impaired ePVS on the ipsilateral side of the stroke and which do not recover post‐stroke are predictive of a greater likelihood and more rapid development of PSE due to severely impaired waste‐clearing mechanisms.

This is illustrated by studies, described in detail above, investigating ePVS in patients with focal epilepsy that indicate that PVS asymmetry is correlated to the SOZ.^62^, ^66^, ^67^

As in generalized epilepsy and neurodegenerative and neuroinflammatory diseases,^28^, ^29^, ^31^, ^32^, ^49^, ^103^ it is thus hypothesized that in PSE and other focal epilepsy conditions, it is relative ePVS asymmetry rather than absolute ePVS number and volume, which is associated with pathology and epileptogenesis.^62^, ^66^, ^67^

3 Longitudinally, a change in the asymmetry rate of ePVS over time is positively correlated with development of PSE.

Most reports of ePVS asymmetry and reduced glymphatic clearance as an underlying mechanism for epileptogenesis are cross‐sectional or retrospective case–control studies.^62^, ^63^, ^66^ However, those studies do not investigate how changes of ePVS over time affect epileptogenesis, and there is a lack of longitudinal studies investigating ePVS dynamics.

Animal studies do indicate that following an AIS, dynamics of ePVS play a role in the remodeling of injured brain tissue, and a link has been found between impaired glymphatic waste clearance and the development of post‐stroke dementia.^104^

While we do not completely understand the ePVS volume and count changes over time, an asymmetry is indicative of pathological mechanisms related to acute injury and epileptogenesis.

Longitudinal clinical studies investigating ePVS dynamics could identify the potential relationship of greater ePVS asymmetry with impaired glymphatic clearance ipsilateral to the stroke in the acute and subacute phase post‐stroke and the increased likelihood of a progression of asymmetry in line with the development of PSE. It is therefore hypothesized that changes in glymphatic clearance in the penumbra of a stroke correlate to the accumulation of excitotoxic glutamate, which further enhances the development of epileptogenic networks.^105^

## 4 POTENTIAL PROBLEMS WITH THE HYPOTHESES

ePVS are a potential biomarker in various neurological and neurodegenerative diseases. However, there is presently insufficient evidence to comprehensively elucidate whether ePVS serve as a cause or a consequence of disease. When employed as a diagnostic tool, discerning its causal relationship may be less critical. However, when considering its potential utility as a treatment target or prognostic marker, it becomes imperative to ascertain whether ePVS is a cause or a consequence. In many cases, for example in AD, ePVS is an unspecific sign of disease activity, which is also seen in overlapping conditions such as vascular dementia and frontotemporal dementia.^103^, ^106^

While ePVS may have limited use in the differential diagnosis of dementias, studies in AD have shown relationships with cognitive decline and studies in MS associations with disease severity.^29^, ^31^ Therefore, ePVS may be a marker of disease severity and sequelae.

Instead of being an independent factor of PSE, ePVS may be a general sign of an injured microvasculature, indicative of comorbidities such as ischemic small vessel disease or general aging. Furthermore, there is a risk of overdetecting similar‐looking pathological features such as lacunar infarcts or white matter hyperintensities (WMH) as ePVS, both common comorbidities in stroke patients.^73^

Furthermore, due to the lack of longitudinal studies, little is known about ePVS characteristics across the lifespan as a global measure of dysfunction. However, in PSE and other focal epilepsy conditions, there are indications that relative ePVS asymmetry in relation to the site of injury, independent of global lifespan ePVS changes is a measure of epileptogenesis. Yet, there is a limited number of studies highlighting the relationship between epilepsy and ePVS. Hence, it is difficult to distinguish between degenerative changes related to epilepsy versus acute changes post‐seizure, and thus, we do not know exactly when these changes happen. It would therefore be necessary to follow patients at different time points post‐stroke to study ePVS dynamics and its relationship with post‐stroke epileptogenesis.

## 5 LIMITATIONS AND FUTURE RESEARCH DIRECTIONS

The concept of ePVS and the glymphatic system and its implication for several brain diseases are relatively new. To date, there is little literature explaining a possible pathophysiological link between the impairment of ePVS and epilepsy. Animal studies have investigated the link between ePVS dysfunction and stroke; however, to our knowledge, very few human studies have been conducted to explain the possible connection between neuroinflammation post‐stroke and the dynamics of ePVS. A handful of retrospective and cross‐sectional studies on PVS function and asymmetry have been conducted, yet longitudinal studies investigating ePVS dynamics and seizure development are lacking.

ePVS has the potential as a novel neuroimaging biomarker of PSE. Novel adaptive automated filtering and segmentation methods to detect and quantify ePVS both in retrospective and prospectively collected cohorts will enable the study of the dynamics of ePVS as a possible predictor for PSE. These methods while presenting a non‐invasive approach compared to invasive studies of ePVS^36^ are still in the developing phase and do not yet follow a standard protocol. We further believe that ePVS in PSE, once thoroughly investigated, could have the characteristics of a risk‐predicting biomarker, with a focus on relative ePVS asymmetry, rather than absolute PVS counts that differentiates it from neurodegenerative conditions. Hence, combined with other clinical and non‐clinical parameters, it could contribute in the development of predictive algorithms for PSE. If successful, this could help identify patients at high risk of developing PSE, who could potentially benefit from a future antiepileptogenesis trial.

## 6 CONCLUSION

ePVSs are known to be asymmetric in focal epilepsy, but it is still to be determined whether or not it is a predictor for PSE. Furthermore, longitudinal studies and imaging across the lifespan are needed to better elucidate the role of ePVS in post‐stroke epileptogenesis.

## AUTHOR CONTRIBUTIONS

Gernot Hlauschek drafted the manuscript. John‐Paul Nicolo, Benjamin Sinclair, Meng Law, Morten Ingvar Lossius Clarissa Yasuda, and Fernando Cendes reviewed and edited the manuscript. Gernot Hlauschek, Morten Ingvar Lossius, Patrick Kwan, and Lucy Vivash conceived the topic. All authors reviewed and approved the final version of the manuscript.

## CONFLICT OF INTEREST STATEMENT

The author Gernot Hlauschek has served as a lecturer for Eisai, unrelated to this study. The author Morten Ingvar Lossius has served as a paid consultant and lecturer for Eisai, UCB, and Arvelle Therapeutics, unrelated to this study. None of the other authors has any conflict of interest to disclose.

## ETHICS STATEMENT

We confirm that we have read the Journal's position on issues involved in ethical publication and affirm that this report is consistent with those guidelines.

## Data Availability

The authors confirm that the data supporting the findings of this study are available within the article, its supplementary materials, and reference list.
